# On the evaluation of synthetic longitudinal electronic health records

**DOI:** 10.1186/s12874-024-02304-4

**Published:** 2024-08-14

**Authors:** Jim L. Achterberg, Marcel R. Haas, Marco R. Spruit

**Affiliations:** 1 Public Health and Primary Care, Health Campus The Hague, Leiden University Medical Center, Albinusdreef 2, Leiden, South-Holland 2333ZA Netherlands; 2 Leiden Institute of Advanced Computer Science, Leiden University, Einsteinweg 55, Leiden, South-Holland 2333CC Netherlands

**Keywords:** Synthetic data, Electronic health records, Longitudinal, Goodness-of-Fit, Privacy risk

## Abstract

**Background:**

Synthetic Electronic Health Records (EHRs) are becoming increasingly popular as a privacy enhancing technology. However, for longitudinal EHRs specifically, little research has been done into how to properly evaluate synthetically generated samples. In this article, we provide a discussion on existing methods and recommendations when evaluating the quality of synthetic longitudinal EHRs.

**Methods:**

We recommend to assess synthetic EHR quality through similarity to real EHRs in low-dimensional projections, accuracy of a classifier discriminating synthetic from real samples, performance of synthetic versus real trained algorithms in clinical tasks, and privacy risk through risk of attribute inference. For each metric we discuss strengths and weaknesses, next to showing how it can be applied on a longitudinal dataset.

**Results:**

To support the discussion on evaluation metrics, we apply discussed metrics on a dataset of synthetic EHRs generated from the Medical Information Mart for Intensive Care-IV (MIMIC-IV) repository.

**Conclusions:**

The discussion on evaluation metrics provide guidance for researchers on how to use and interpret different metrics when evaluating the quality of synthetic longitudinal EHRs.

## Introduction

Synthetic data is an emerging method to mitigate privacy concerns when dealing with sensitive personal information [1]. Domains with a large amount of sensitive information, like Electronic Health Records (EHRs) in healthcare, stand to gain the most from this. However, generating high-quality synthetic EHRs is a difficult task for multiple reasons. For example, EHRs potentially span multiple modalities including static demographics and attributes, longitudinal measurements on health factors, clinical text notes, and images [2]. EHRs are commonly used to provide evidence for a wide variety of research topics, like population health [3], risk prediction [4], and medical imaging [5]. In this research we focus on longitudinal EHRs: a combination of static patient attributes and varying-length sequential health measurements, of mixed numerical and categorical data types.

Longitudinal EHRs pose many challenges for which some may be mitigated by employing synthetic data. Firstly, personal information contained in EHRs may hinder their usage in research due to ethical or privacy-related concerns [6]. Synthetic EHRs may mitigate these concerns, since they are ideally untraceable to real individuals [7]. Next to research applicability, secondary applications may be available in educational contexts. Secondly, longitudinal EHRs are often costly to collect since many variables have to be collected over extended periods of time [8]. Through synthetic data generating techniques additional records may be generated at a highly reduced cost. This may be especially helpful in scenarios where data requirements are large [9]. Lastly, EHRs have been shown to potentially induce biases [10, 11]. By generating synthetic records for underrepresented groups, biased datasets can be rebalanced. This may improve outcome equality across subgroups when EHRs are used in downstream tasks [12]. In this research, we mainly focus on the usage of synthetic EHRs instead of real EHRs when these cannot be utilized due to privacy concerns.

Synthetic data can be defined as data sampled from a generative model, designed to mimic the properties of real data [13]. Here, generative models can be rule-based, based on known probability functions, or based on learning and sampling from the latent data space [14]. Recent advancements in generative modelling bring forth model architectures which are able to learn and generate synthetic samples for almost any data structure [15]. Likewise, many model architectures have been developed which are able to generate synthetic longitudinal EHRs [16–18]. However, in current research there is a lack of focus on how to properly evaluate the quality of synthetic longitudinal data. Employed metrics should account for the temporal aspect of the data, and inform the researcher on if and how the generating model is failing in an interpretable manner - for example mode collapse or mode invention [19]. This is also the problem this research focuses on, namely providing recommendations on evaluation metrics suitable for longitudinal synthetic data, whilst extensively discussing their strengths and weaknesses. This way, we aim to provide guidance for researchers interested in employing synthetic longitudinal EHRs.

To this end, we first discuss how to use t-distributed Stochastic Neighbour Embedding (tSNE) [20] and Uniform Manifold Approximation and Projection (UMAP) [21] in combination with Dynamic Time Warping (DTW) [22] to effectively visualize longitudinal EHRs. This way we provide an evaluation method which accounts for temporal structure (through DTW), whilst providing an intuitive assessment of real to synthetic EHR similarity.

Secondly, we discuss how a Recurrent Neural Network (RNN) classifier can be used to compare synthetic and real distributions whilst accounting for temporal correlations in the data. We show that this metric requires some care however, since results may be influenced not only by whether real and synthetic data are indeed similar, but also by whether the choice of classifier is appropriate.

Next, we discuss how to assess real-world utility of synthetic longitudinal EHRs by comparing performance of synthetically versus real trained machine learning models in clinical tasks. Lastly, we recommend to assess privacy risk of disclosing synthetic EHRs by performing an Attribute Inference Attack (AIA) [23] on several sensitive patient attributes. If successful, the AIA exposes the risk of real sensitive attributes being inferred by malicious parties from synthetic data.

We support the discussion on our recommended metrics by applying them on synthetic longitudinal EHRs generated from a dataset from the Medical Information Mart for Intensive Care-IV (MIMIC-IV) repository [24], using models from open-source libraries |Synthetic Data Vault|^1^ [25] and |Gretel-Synthetics|^2^. Both the data and the methods used to generate and evaluate synthetic data are publicly available, and the code to reproduce our results is available via our public GitHub repository^3^.

## Background

### Longitudinal EHRs

Generating longitudinal instead of row-summarized EHRs provides a rich data representation required in a wide variety of research and real-world applications. Sequential patterns present in EHRs provide additional information for example in early disease detection [26], disease progression modelling [27, 28], and mortality prediction [29, 30]. Furthermore, longitudinal synthetic datasets can be used to construct a variety of simpler cross-sectional synthetic datasets for other purposes.

### Synthetic data evaluation

When evaluating synthetic data three aspects are typically investigated [31]:

- Fidelity: resemblance to real data
- Utility: usefulness in real-world tasks
- Privacy risk: risk of disclosing real sensitive information

#### Fidelity

Regarding synthetic data fidelity, we can differentiate between feature-wise similarity, and similarity across features and the sequential (or other) dimension. Feature-wise similarity can be investigated through descriptive statistics, plots, statistical tests, or other metrics. Descriptive statistics provide a first sanity check on whether high-level statistics like mean, variance, and range, are similar. Furthermore, feature-wise drift measures [32] and Goodness-of-Fit (GoF) tests like Kolmogorov-Smirnov (KS) [33] provide statistical confidence on whether feature distributions of real data are accurately captured in synthetic data.

To further evaluate synthetic data fidelity, plots of low-dimensional projections can be provided using dimensionality reduction algorithms [34]. This way, compressed and intuitive representations of similarity between synthetic and real data in the original feature space are provided.

Although they provide intuition, low-dimensional projection plots do not provide a clear numerical representation of synthetic and real distributional similarity. For this purpose, a classification model can be trained to discriminate synthetic from real samples [16, 35, 36]. Here, high accuracy on a test set indicates a classifier easily distinguishes synthetic from real samples, implying their distribution is different. Accuracy close to 50% implies the opposite - that the synthetic and real data distribution are similar. Other metrics exist which are based on this method, like the propensity score Mean Squared Error [37].

#### Utility

Synthetic data utility is typically assessed by investigating whether it can be used instead of real data in common tasks whilst retaining performance. Here, we compare performance of models trained on synthetic and real data when tested on a real test set - also called the Train Synthetic Test Real approach (TSTR) [38]. If performance is similar, synthetic data reflects complexities of real data necessary for commonly performed tasks. Then, if privacy metrics indicate adequate privacy preservation, it can be used in practice instead of real data to preserve privacy. This approach is widely used to assess the utility of synthetic medical data [16, 34, 35].

#### Privacy risk

To evaluate privacy-preserving capabilities of synthetic EHRs, many metrics exist. Differential privacy [39] provides mathematical guarantees on individual-level privacy, but is often difficult to interpret in a practical setting [40]. Membership inference attacks [41] indicate whether third parties can infer which real individuals were used in training the synthetic data generating model. However, this requires a subset of patients to already be known to the attacker. In this research, we assess privacy risk by performing an AIA [23]. Specifically, an AIA where the attacker has access to a set of incomplete EHRs without access to the synthetic data generating model - and tries to infer the missing sensitive information.

## Limitations of existing works

There are many limitations to synthetic data generating techniques, which have previously been attempted to overcome. Examples include mode collapse [42, 43], training instability [44, 45], imbalanced training data [46, 47], and more. However, since this research focuses on limitations of synthetic data evaluation rather than generation, this is what the rest of this section focuses on.

To evaluate synthetic data fidelity, Pei et al. [34] provide plots of low-dimensional projections using tSNE. However, for varying-length sequences, computing sample distances required for projection algorithms like tSNE is non-trivial: it is not directly clear how datapoints within sequences map to each other. For this purpose, we propose to use DTW to first align varying-length sequences.

Other methods exist to project data to a low-dimensional space with the purpose of visualization. Gisbrecht and Hammer [48] provides a review of similarities and differences between popular methods, and argues that they can be categorized according to (among other things) whether they are linear, non-linear, parametric or non-parametric. As we assume no prior knowledge on the data generating process and the data might exhibit complex non-linear patterns, we opt for a non-linear and non-parametric method. For this, tSNE is an apt candidate. Interestingly, the authors of UMAP [21] claim it is potentially better at visualizing global data structure than tSNE, so we include UMAP (also non-linear and non-parametric) next to tSNE for comparison.

The tSNE and UMAP algorithm have some notable limitations however. Firstly, both are stochastic algorithms which may produce different outputs depending on initialization and hyperparameters [49, 50]. To account for this, we can show outputs for different values of influential hyperparameters. Secondly, the output of both algorithms relies heavily on the distance metric chosen to compute distances between samples in the dataset. Studies like Smets et al. [51] have shown the sensitivity of tSNE and UMAP to different distance metrics. Thus, choosing an apt distance metric and understanding its limitations is vital.

To provide a numerical representation of synthetic to real similarity, Li et al. [16], Lee et al. [35], Kaur et al. [36] train a classifier to discriminate synthetic from real samples and report the accuracy on a test set. However, we will show that reporting only the accuracy metric does not provide the full picture when evaluating synthetic records. This metric might be an oversimplification of distributional similarity, and analyzing classifier predictions through plots or statistical tests may be useful.

Kaur et al. [36], Choi et al. [52], Goncalves et al. [53] assess risk of attribute inference in tabular synthetic records. To our knowledge, no literature exists on assessing this risk in longitudinal health records. AIAs in longitudinal records require appropriate inference models, which are able to capture sequential correlations.

## Methods

### Dataset description

We select a dataset of longitudinal EHRs to illustrate our discussion on evaluation metrics. The dataset was obtained from the MIMIC-IV repository [54] on Physionet [55] (version 2.2), a freely available resource consisting of de-identified EHRs from the Beth Israel Deaconess Medical Center in Boston, Massachusetts, between 2008 and 2019. We select patients who suffered ischemic heart disease, selecting ICD-9 (International Classification of Diseases-9) code sequences and static patient attributes *age*, *race*, *gender*, and *deceased* (whether a patient passed away in-hospital). Since there are over 13,000 possible ICD-9 codes, we encode diagnoses by their ICD-9 chapter^4^ to reduce computational complexity. Since the chapters *complications of pregnancy, childbirth, and the puerperium*, *congenital anomalies*, and *certain conditions originating in the perinatal period* are extremely rare in patients with ischemic heart disease, we omit these diagnoses completely.

Regarding missing data, this plays a role mostly in patient attributes. In sequences of diagnoses codes, missingness shows as a sequence being of different length than it would otherwise be - although it cannot be said if values are missing in a specific sequence. Variable-length sequences are handled by using appropriate methods such as DTW. In patient attributes, there is 11% missingness in *race*, and no missingness in *age* and *gender*. Since values might be missing not-at-random, we encode missing values as a separate category (*unknown*).

The final dataset contains 18,245 patients, with 4 static attributes and a single diagnoses sequence with length between 5 and 37 each. Note that this dataset of longitudinal EHRs is less complex than required for many real-world clinical tasks. ICD-9 codes are encoded by their chapter, and only a small set of patient attributes and sequential health data is selected. This is because this dataset is only used for illustrative purposes, to support the discussion on evaluation metrics for synthetic EHRs.

### Synthetic data generating models

We generate synthetic patient data using two distinct deep learning models contained in open-source software libraries. Firstly, a Generative Adversarial Network (GAN) [56] with DoppelGANger^5^ [57], and secondly a Conditional Probabilistic Auto-Regressive network^6^ (CPAR) [58]. The DoppelGANger implementation used does not provide support for mixed-length sequences, so we implement a mask following Lin et al. [57] in the package. Both models generate data in two steps, by generating static attributes followed by sequential data conditional on generated attributes. This allows the models to capture the relationship between patient attributes and the progression of diagnoses codes. For both DoppelGANger and CPAR we generate the same number of records as present in the real dataset.

Note that other models have been developed which are able to generate EHRs with static attributes and sequential data. We opt for DoppelGANger and CPAR since they are contained in easy-to-use open-source libraries, promoting reproducibility of this research. Generating synthetic data of the highest quality is not the goal here, as we are providing a discussion of and recommendations on evaluating the quality of synthetic longitudinal EHRs. Other notable methods include Li et al. [16], Theodorou et al. [17], Mosquera et al. [18], Lee et al. [35], where we recommend using deep generative models like GANs and VAEs in the case of high-dimensional datasets. Since, these methods reduce the complexity of the learning task to a lower-dimensional continuous latent space.

### Evaluating fidelity

#### Descriptive statistics

The first step in evaluating synthetic data fidelity is investigating descriptive statistics. We evaluate boxplots of numerical variables, and relative frequencies of categorical variables. For sequential features we compute these statistics at each step.

#### Low-dimensional projections

To intuitively assess synthetic to real data similarity, we visualize synthetic and real multivariate samples in two dimensions. If synthetic and real samples mostly overlap in the plot, this indicates they are similar. Additionally, this method may indicate whether mode collapse is present, which is the case when synthetic samples are realistic but of very low variety. In a plot, this may show as synthetic samples clustered into one or more dense clouds, instead of following the same dispersion as real datapoints.

Visualizing multivariate samples in two dimensions requires an algorithm which can adequately project multivariate samples to two dimensions. We use tSNE and UMAP for this purpose. Both algorithms compress datasets by constructing datapoints in a low-dimensional space, which exhibit similar divergence between datapoints as the original data, according to some metric. This way, they aim to preserve the overall structure of the data, even after major compression of the feature space [20, 21]. An important hyperparameter in both algorithms is the number of neighbours (called perplexity in tSNE), which controls the amount of datapoints which are considered when calculating divergences.

Although there are similarities, many differences exist between tSNE and UMAP. For example, tSNE calculates divergence between *all* datapoints using Kullback-Leibler divergence, whereas UMAP calculates divergence only between *k*-nearest neighbours and uses cross-entropy. UMAP intends to improve upon tSNE in terms of speed and quality [21], but some research claims differences in quality are mainly due to choice in initialization and can thus easily be mitigated [50].

However, when considering longitudinal datasets, calculating divergence between datapoints in tSNE and UMAP adds another dimension of difficulty. For sequential features of variable length, standard metrics do not directly apply since it is unclear how datapoints between samples map to each other. For this reason we first apply the DTW algorithm, which finds a mapping between datapoints of two sequences which minimizes the total divergence between them - subject to some conditions. The mapping starts and ends at the start- and end-point of both samples, and is monotonic and continuous [22].

Lastly, in order for the DTW algorithm to find a mapping between datapoints of two sequences, it requires choosing an appropriate divergence metric. Since we consider features of mixed data types, we use Gower distance [59], which is fit for this purpose.

Since the DTW algorithm returns the cumulative Gower distance over aligned sequence steps, this needs to be scaled to a similar range before averaging static and sequential feature distances. We use the |DtwParallel| package to execute the DTW algorithm, which scales distances with the geometric mean of sequence lengths. This choice can be justified over the use of arithmetic mean-based scaling, since it ensures that sequence length variability is penalized more heavily.

Finally, it should be noted that using Gower distance as a divergence metric significantly impacts results. Continuous feature distances are at most 1, but only for the most dissimilar case. However, categorical feature distances are 1 in case of any difference - so possibly for many cases. For this reason, differences in categorical features tend to overshadow differences in continuous features when measured through Gower distance.

#### Goodness-of-Fit

The next step in evaluating synthetic data fidelity, is a numerical assessment of synthetic to real data GoF. In other words, measuring the similarity between the synthetic and real data density. However, some method to approximate these densities is required, since they are (usually) intractable. This is often framed as a classification task to discriminate synthetic from real samples, where accuracy close to 50% signifies densities are similar [16, 35, 36]. Note that this closely relates to classification-based GoF testing as in Friedman [60], although synthetic data literature omits explicit testing. In this section we describe strengths and weaknesses of this metric, and how it can be used to statistically test whether the synthetic and real data density are the same in the scenario of longitudinal datasets.

Firstly, comparing synthetic to real data density through this classification task may in some cases be an oversimplification of the problem. Since, we can show mathematically that it implies the following: the entire multivariate data distribution can be encoded as a simple univariate binary feature, for which we approximate its density using a classifier. Below follows the mathematical proof.

Let $\textbf{X}_{\textbf{R}}$ be the original dataset of real samples, $\textbf{X}_{\textbf{S}}$ a generated synthetic dataset, and pooled dataset $\textbf{X} = \textbf{X}_{\textbf{R}} \cup \textbf{X}_{\textbf{S}}$. Since densities $p(\textbf{X}_{\textbf{R}}),p(\textbf{X}_{\textbf{S}})$ are intractable, we encode them with the univariate binary feature $\textbf{z} = \textbf{1}_{\textbf{X}_{\textbf{S}}}: \textbf{X} \rightarrow \{0,1\}$ and approximate its posterior $p(\textbf{z}|\textbf{X})$ with $q_{\lambda }(\textbf{z})$ through variational inference [61]. Here, we choose $q_{\lambda }$ as some machine learning model where $\lambda$ are its parameters. Now, we can optimize for $\lambda$ when minimizing the Kullback-Leibler divergence [62] between the true and approximated posterior of $\textbf{z}$:

1 $$\begin{aligned} q_{\hat{\lambda }}(\textbf{z}) & = \arg \underset{\lambda }{\min }\ \text {KL}(q_{\lambda }(\textbf{z}) \Vert p(\textbf{z}|\textbf{X})) \nonumber \\ & = \arg \underset{\lambda }{\min }\ - \sum \limits _i z_i \log \left( \frac{z_i}{q_i}\right) \nonumber \\ & = \arg \underset{\lambda }{\min }\ \sum \limits _i - \log {q_i} \end{aligned}$$,

where $z_i$ are binary labels and $q_i$ label predictions from $q_{\lambda }(\textbf{z})$. This is equivalent to optimizing a binary classifier $f_{\theta }$ for minimal cross-entropy between true labels and predictions:

2 $$\begin{aligned} f_{\hat{\theta }}(\textbf{z}|\textbf{X}) & = \arg \underset{\theta }{\min }\ \text {H}(f_{\theta }(\textbf{z}|\textbf{X}),p(\textbf{z}|\textbf{X})) \nonumber \\ & = \arg \underset{\theta }{\min }\ -\sum \limits _i z_i \log q_i \nonumber \\ & = \arg \underset{\theta }{\min }\ \sum \limits _i - \log q_i \end{aligned}$$,

such that approximating $p(\textbf{z}|\textbf{X})$ can be framed as a simple binary classification task, since the optimization problems in Eqs. (1) and (2) are equivalent. In this case, note that we make a continuous approximation of the binary latent variable on the (0,1) interval. Lastly, note that training, optimizing and reporting results in the classification should be done with independent train, validation and test sets. This is to avoid overfitting the training data, and results in a more reliable approximation of $p(\textbf{z}|\textbf{X})$.

Encoding the multivariate synthetic and real distribution as univariate binary labels has some clear advantages. It allows for relatively simple approximation through classification, and consequently a univariate representation of the multivariate distributions. This allows for intuitive visualization to inspect model failures such as mode collapse, but also explicit GoF testing of the latent distribution. When $H_0: q_{\lambda }(\textbf{z}_{\textbf{S}})=q_{\lambda }(\textbf{z}_{\textbf{R}})$ is rejected after performing a univariate GoF test like the KS test, we can be certain also $H_0: p(\textbf{X}_{\textbf{S}})=p(\textbf{X}_{\textbf{R}})$ is rejected. By explicit GoF testing, we can provide statistical confidence on whether the synthetic and real data density are similar.

However, when $H_0: q_{\lambda }(\textbf{z}_{\textbf{S}})=q_{\lambda }(\textbf{z}_{\textbf{R}})$ holds it does not necessarily follow that $H_0: p(\textbf{X}_{\textbf{S}})=p(\textbf{X}_{\textbf{R}})$ also holds. Firstly, the chosen family of densities (machine learning classifiers) $q_{\lambda }$ might not be suitable. Secondly, the compression of the multivariate dataset into a univariate binary feature $\textbf{z}$ might be too simplistic. This metric should thus be approached with care.

To mitigate the risk of binary classification being an oversimplification of the problem, an option is to let $\textbf{z}$ be a *multidimensional* Gaussian and approximate $p(\textbf{z}|\textbf{X})$ using a Variational Auto-Encoder (VAE) [63]. Then, testing $H_0: q_{\lambda }(\textbf{z}_{\textbf{S}})=q_{\lambda }(\textbf{z}_{\textbf{R}})$ can be done through a multivariate GoF test. This way, the added dimensions allow for capturing more intricate differences between the synthetic and real distribution.

In the scenario of longitudinal datasets, the classifier should capture differences across the time dimension as well. An appropriate classifier which can handle mixed-length sequences of mixed data types, is an RNN classifier. This is the classifier we use to analyze classifier predictions and perform the KS test to test $H_0: q_{\lambda }(\textbf{z}_{\textbf{S}})=q_{\lambda }(\textbf{z}_{\textbf{R}})$.

Whenever we use an RNN we specifically mean a neural network with at least one layer containing Gated Recurrent Units (GRU) [64] to process sequential inputs. Long Short-Term Memory (LSTM) cells [65] are likely not necessary since sequences are relatively short [66].

### Evaluating utility

Typical clinical tasks involving patient attributes and diagnoses sequences are, among other things, in-hospital mortality prediction using RNNs [30, 67] and next-step diagnoses prediction using RNNs and attention networks [27, 28, 68]. To assess utility of the generated synthetic EHRs, we can compare performance in these respective tasks with the TSTR approach.

It should be noted that we do not aim for the best performance possible here. The dataset was not selected with a specific use case in mind, and serves an illustrative purpose to support the discussion on evaluation metrics. For showcasing the TSTR approach the comparison in performance is key, the level of performance less so - as long as the algorithm has at least some predictive power. When aiming for the best performance possible in these tasks, we recommend to include additional features like socioeconomic status, physiological measurements and medications, which have empirically shown to be important [30, 67, 69–71] - next to omitting chapter encoding of ICD-9 codes.

### Evaluating privacy risk

To assess risk of attribute inference, we train an RNN to predict sensitive attributes from all non-sensitive features. Here, we take every possible combination from the feature set {*age*,*gender*,*race*} as sensitive target attributes - so 7 sets in total. Every feature not in the set of target attributes, is used as a non-sensitive input feature in that iteration.

We assess the risk of attribute inference through the predictive accuracy of this AIA. Here, we focus on interpretable metrics such as accuracy and Mean Absolute Error (MAE) to assess the potential privacy risk in a real-world setting.

## Results

As mentioned in Synthetic data generating models section, the generated synthetic datasets contain the same number of records as the real dataset: 18,245. The datasets contain 4 static attributes *age*, *gender*, *race*, *deceased*, next to diagnoses sequences consisting of anywhere between 5 and 37 diagnoses per patient.

### Fidelity

To evaluate fidelity of synthetic EHRs, we assess descriptive statistics, two-dimensional plots through tSNE and UMAP, and a classification-based GoF metric.

#### Descriptive statistics

**Fig. 1:**
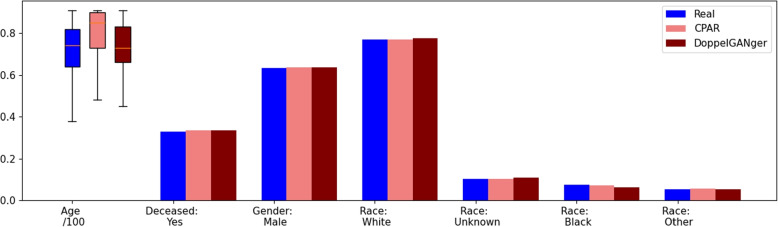
Descriptive statistics of static features (Shows numerical feature *age* divided by 100 and categorical features by frequency)

Figure 1 shows descriptive statistics of static features *age, gender, deceased*, and *race* for real EHRs and synthetic EHRs generated from CPAR and DoppelGANger. Overall, the frequency of the categorical features from synthetic EHRs closely match those of real EHRs. For the *age* feature, synthetic EHRs from DoppelGANger more closely match the real EHR statistics than those generated from CPAR.

**Fig. 2:**
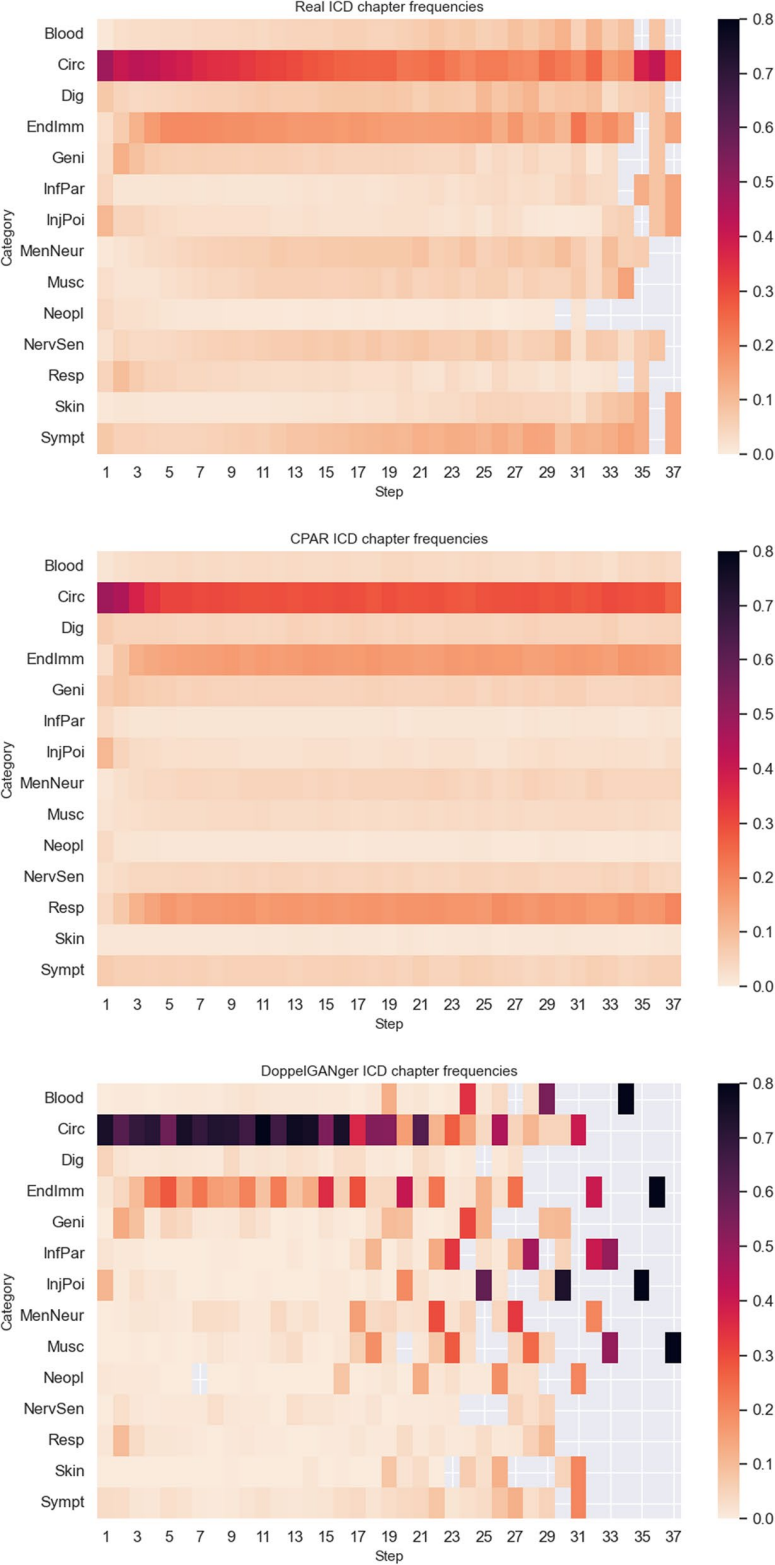
Descriptive statistics of sequential features (Heatmaps show the frequency of ICD chapters at each sequence step)

Figure 2 shows descriptive statistics of the sequential ICD chapters for real EHRs and synthetic EHRs generated from CPAR and DoppelGANger. For synthetic EHRs from CPAR, ICD chapter frequencies are generally well captured at each step - except for *diseases of the respiratory system* (*Resp*), which CPAR overestimates. For synthetic EHRs from DoppelGANger, the variability of generated ICD chapters is much lower than in real EHRs. Although *diseases of the circulatory system* (*Circ*) are the most prevalent in real EHRs, DoppelGANger overestimates the frequency of this diagnosis - this is a potential sign of mode collapse.

#### Low-dimensional projections

**Fig. 3:**
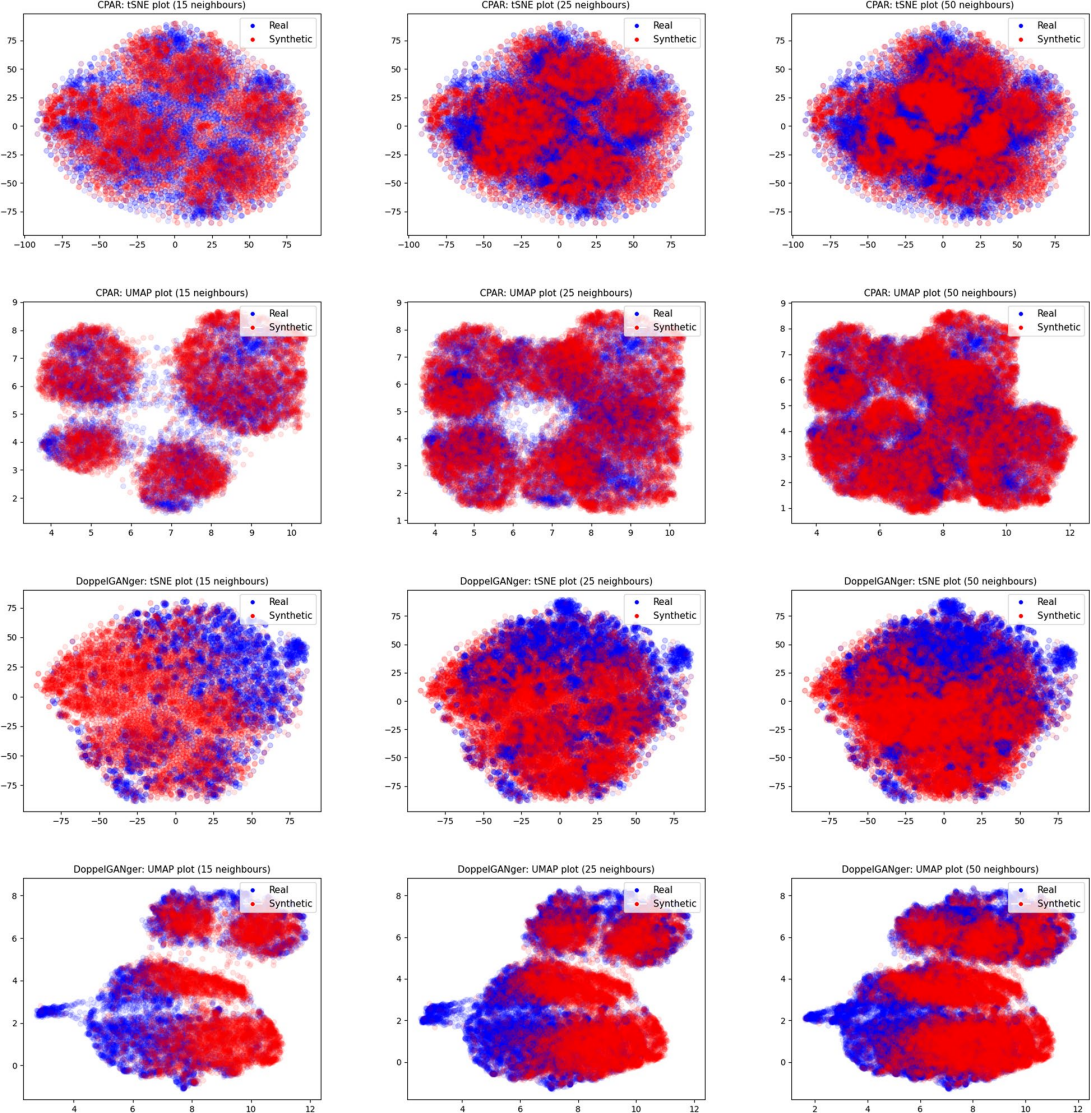
Scatter plots of projections by tSNE and UMAP

Figure 3 shows scatter plots of low-dimensional projection plots made using tSNE and UMAP. Since the number of neighbours - or for tSNE, perplexity - chosen in the corresponding algorithm can highly influence the projections, we plot them for three different values (15, 25 and 50).

Figure 3 shows that for both CPAR and DoppelGANger, synthetic samples are projected as clouds of points *within* larger clouds of real samples. This is line with the idea that both CPAR and DoppelGANger output somewhat realistic samples, but of too small variety - indicating mode collapse. This issue seems especially prevalent in samples generated from DoppelGANger.

#### Goodness-of-Fit

To evaluate $H_0: p(\textbf{X}_{\textbf{S}})=p(\textbf{X}_{\textbf{R}})$ we encode the multivariate distributions as latent feature $\textbf{z}=\textbf{1}_{\textbf{X}_{\textbf{S}}}: \textbf{X} \rightarrow \{0,1\}$ and approximate $p(\textbf{z}|\textbf{X})$ through classification with an RNN, to test $H_0: q_\lambda (\textbf{z}_{\textbf{S}})=q_\lambda (\textbf{z}_{\textbf{R}})$. For the RNN, the hidden layers consist firstly of separate dense units and GRUs for static and sequential input respectively, followed by a joint fully-connected layer.

On 10-fold cross-validated test sets we achieve average classification accuracy for CPAR: 0.83 (0.01), DoppelGANger: 0.89 (0.02), with standard deviation between brackets. Additionally, we find $H_0: q_\lambda (\textbf{z}_{\textbf{S}})=q_\lambda (\textbf{z}_{\textbf{R}})$ does not hold ($p<.000$) for both.

**Fig. 4:**
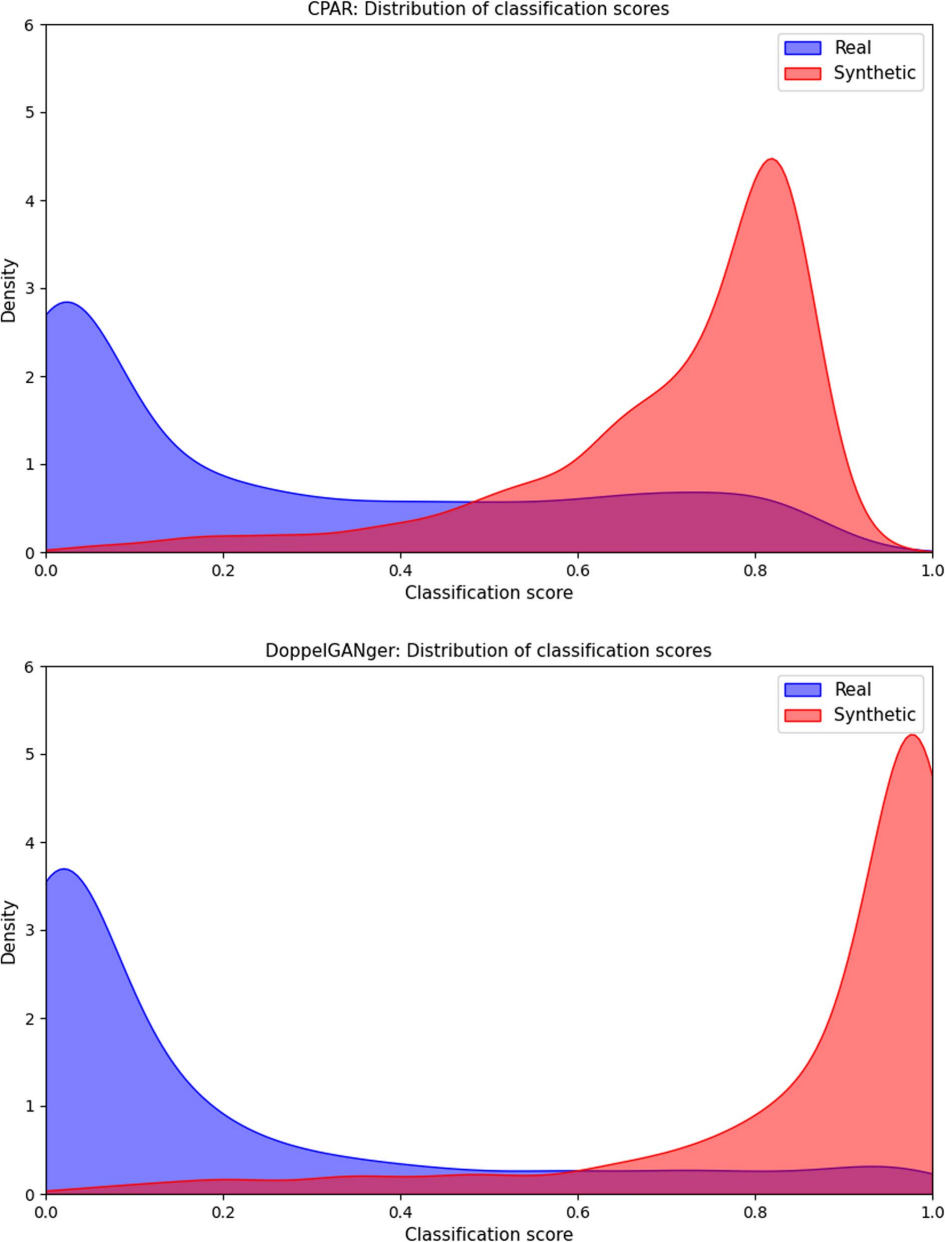
Kernel density estimate plot of classifier predictions (Example of a kernel density estimate plot of classifier predictions from the first of 10-fold cross-validated results, discriminating synthetic from real samples. Bandwidth is selected using Scott’s rule [72])

The distributions of classifier predictions in Fig. 4 show the generated samples from both CPAR and DoppelGANger are too simplistic, even when compressed univariately by a classifier. The plots indicate mode collapse, since the synthetic compressed distributions seem collapse to one of the (smaller) modes of the real compressed distribution. This is in line with results from descriptive statistics and low-dimensional projection plots.

### Utility

To evaluate synthetic EHR utility, we compare performance in the TSTR approach in clinical tasks mortality prediction and next-step diagnoses prediction.

#### Mortality prediction

We evaluate in-hospital mortality predictions using an RNN (similar architecture as in Goodness-of-Fit section), from ICD chapters and static patient attributes.

On 10-fold stratified cross-validated test sets we achieve average Area Under Curve (AUC) [73], when trained on real and synthetic data respectively, for CPAR: (0.57 (0.01), 0.56 (0.01)) and for DoppelGANger: (0.58 (0.01), 0.57 (0.01)), with standard deviation between brackets.

We achieve poor AUC scores (< .60) in mortality prediction, even when using real data. This comes as no surprise, since our dataset was not selected with the aim of predicting mortality - diagnoses are encoded by their chapter, and we omit clinically import features like patient socioeconomic status and physiological measurements and medication. Nonetheless, the RNN consistently achieves AUC > 0.55 across all folds and thus has some (small) predictive power. This allows us to use these models as a comparison between real and synthetic training data, in which the difference in resulting performance is more important than the predictive power by itself.

The synthetically trained models achieve similar AUC as the models trained on real data - and thus, synthetic EHR could replace real EHRs in mortality prediction using RNNs. In practice, this should be validated with a more complete synthetic dataset, containing more features relevant in mortality prediction.

#### Next-step diagnoses prediction

We evaluate next-step diagnoses predictions using an RNN (similar architecture as in Goodness-of-Fit section), from previous-step diagnoses and static patient attributes.

On 10-fold cross-validated test sets we achieve average accuracy, when trained on real and synthetic data respectively, for CPAR: (0.31 (0.01), 0.31 (0.00)) and for DoppelGANger: (0.31 (0.01), 0.31 (0.00)), with standard deviation between brackets. Again, accuracy when trained on real and synthetic data is similar, indicating good utility. However, the overall accuracy is poor due to the simplistic nature of the dataset, albeit in a 14-class classification problem. In practice, this should also be validated with a more complete synthetic dataset, just as in mortality prediction.

### Implications of fidelity and utility

In the results on fidelity and utility, we mention several times that the output indicates mode collapse in the generated synthetic datasets. This means the synthetic dataset contains many realistic samples of low variety. Depending on the context in which the synthetic EHRs are used, this may or may not be a large issue.

Synthetic data can be used to inform a public which is not allowed access to real data, on what a realistic dataset looks like. An example of this is an educational context, where (medical) students learn through examining synthetic data [74]. Here it is crucial that minimal mode collapse occurs, as students should learn about the full range of possible records. Relating that to this research, using models like GANs which may exhibit mode collapse might not be recommended.

Another context is using a synthetic dataset in a specific modelling task, for example in answering a research question or developing a data-driven application [75]. In this context, mode collapse may be less of an issue. Since, as shown in Xing et al. [76], synthetic data does not always have to have high variety to have high utility in specific tasks. When a low variety of synthetic samples still exhibit realistic patterns required in a task, a dataset where mode collapse occurred may still be adequate. However, it is still possible that a specific task requires a wide variety of samples, so this should be examined on a case-by-case basis. As we found comparable utility of synthetic data to real data, mode collapse may not be a large issue in this work.

### Privacy risk

To evaluate privacy-preserving capabilities of synthetic EHRs, we assess the risk of inference of sensitive real information through an AIA.

#### Attribute inference attack

We evaluate real sensitive feature ({*age, gender, race*}) predictions using an RNN (similar architecture as in Goodness-of-Fit section) trained on synthetic EHRs.

Table 1 shows average results from the 10-fold cross-validated AIA. For the attribute *age*, the AIA has an MAE of over 13 years on a mean age of 71 years. This is likely not considered a privacy risk in practice, as this error is quite large. For the attribute *gender*, the AIA has an accuracy of close to 50% - indicating the attack is only slightly more accurate than random guessing. Lastly, for the attribute *race*, the AIA is not able to perform much better than exclusively voting for the majority class (*white*). So, the AIA has again little discriminative power on an individual level, and thus seems to pose little privacy risk. This is the case for synthetic samples from both CPAR and DoppelGANger.

**Table 1:** Accuracy of attribute inference attack

Feature set	Feature:Metric	CPAR	DoppelGANger
{Age}	Age:MAE	13.39 (1.15)	13.73 (1.17)
{Gender}	Gender:Accuracy	0.56 (0.01)	0.56 (0.01)
{Race}	Race:Accuracy	0.75 (0.02)	0.76 (0.03)
{Age, Gender}	Age:MAE	13.81 (1.34)	13.71 (1.34)
	Gender:Accuracy	0.56 (0.01)	0.56 (0.01)
{Age, Race}	Age:MAE	14.03 (1.45)	13.88 (1.39)
	Race:Accuracy	0.76 (0.02)	0.77 (0.02)
{Gender,Race}	Gender:Accuracy	0.54 (0.01)	0.56 (0.01)
	Race:Accuracy	0.76 (0.01)	0.77 (0.02)
{Age,Gender,Race}	Age:MAE	15.91 (1.81)	14.18 (1.57)
	Gender:Accuracy	0.57 (0.01)	0.57 (0.01)
	Race:Accuracy	0.73 (0.01)	0.77 (0.02)

Note: First column denotes target features of the inference attack, second column denotes the metric corresponding to the feature, last two columns provide average results after 10-fold cross-validation with standard deviation between brackets

## Discussion

This research provides a discussion on methods to evaluate the quality of synthetic longitudinal EHRs, which should guide researchers in the future. We observed that previously used methods often fail to address specific weak points, like mode failures and the restrictive assumptions under which results hold.

Firstly, two-dimensional plots constructed through tSNE and UMAP - and DTW to align variable-length sequences - provide an intuitive visualization of synthetic to real data similarity. We recommend to use dimensionality reduction algorithms like tSNE and UMAP, in combination with DTW when handling longitudinal data, when there is suspicion of mode collapse or other related issues in synthetic data generation.

Furthermore, we recommend to assess the distribution of classification scores to assess synthetic to real GoF, instead of only relying on average classification accuracy. This way, we can test equivalence between the latent synthetic and real distribution, and univariately assess the latent distribution - for example for mode collapse. Moreover, we recommend to take any positive conclusions from this metric with a grain of salt, as it places very restrictive assumptions on the latent dimensionality of the synthetic and real distribution.

Regarding synthetic data utility, the pragmatic TSTR approach is popular for good reason. It is the closest one can get to evaluating usefulness in a specific real-world setting. Since this approach is already popular, we place little emphasis on it in this research, but include it to show it is good practice.

Instead of mathematical privacy guarantees like in differential privacy, the results from our AIA provide a measure of privacy risk in a real-world setting with malicious attackers. However, we perform the AIA ourselves, and it is possible that an attacker is able to construct a more powerful AIA model. We try to mitigate this risk by using flexible and powerful neural networks. Additionally, in real-world settings, the acceptable amount of privacy risk depends on both use case and the specific dataset sensitivity.

## Practical implications

To guide researchers on which evaluation metrics proposed in this research should be considered, it is vital to consider the specific context in which synthetic EHRs are used. Phenomena like mode collapse are especially hurtful in situations where realism is key, like education. Thus, in these contexts metrics which can expose these phenomena, like visualization through tSNE, UMAP and classifier prediction plots, should be considered. On the other hand, in contexts where performance in a specific modelling task is considered, utility metrics like TSTR are more important. Since, utility may potentially be high even when fidelity is not.

## Conclusions

This research provides a discussion on methods to evaluate synthetic longitudinal EHRs to guide researchers.

Next to descriptive statistics, we utilized tSNE and UMAP to visually assess synthetic to real data similarity, due to their ability to realistically display local and global structure of a dataset in low dimensions. Here, we first use DTW to compute distances between variable-length sequences. When applied to synthetically generated datasets, we found that tSNE and UMAP were able to visualize mode collapse in synthetic data generating models.

Next, for numerical assessment, we discussed the use of a classifier to discriminate synthetic from real samples. When applied to synthetic datasets, the classifier shows that synthetic and real samples are easily separable, and plots of classification scores again indicate mode collapse.

However, we also showed that this metric has some clear drawbacks, namely that it places restrictive assumptions on how the real and synthetic multivariate dataset can be compressed. Further research should investigate methods to alleviate these restrictive assumptions, while still providing a clear numerical representation of the GoF. A possible avenue of exploration is using a VAE to embed synthetic and real data to a multidimensional Gaussian latent space, to subsequently test equivalence of the latent distributions with a multivariate GoF test.

Also, we evaluate real-world utility of synthetic EHRs through performance in the TSTR approach in the clinical tasks mortality prediction and next-step diagnoses prediction. Since comparable performance was achieved from synthetic and real datasets, we conclude that synthetic datasets retain adequate utility. However, although comparable, overall performance was quite low and the methods we use should be validated in future research with larger datasets containing more variables.

Lastly, AIAs on real sensitive information using synthetic datasets indicated little risk of inferring real sensitive information from synthetic data. This generally indicates little risk of leaking sensitive information through synthetic data. However, other privacy-related metrics exist (such as membership inference) which should be explored in future research.

This research offers recommendations on metrics suitable for evaluating synthetic longitudinal EHRs. These recommendations can serve as a valuable resource for researchers and data scientists in the healthcare sector who are involved in generating synthetic records. In addition to these recommendations, the comprehensive discussions on the strengths and weaknesses of the metrics facilitate accurate interpretation, thereby supporting the appropriate adoption of synthetic data in healthcare.

## Data Availability

The dataset is available through the MIMIC-IV repository, for which access for research purposes can be requested through https://physionet.org/content/mimiciv/. Furthermore, code used for analyses is available at https://github.com/JimAchterbergLUMC/SynLongEHR. We used Python 3.10.

## Abbreviations

AIA: Attribute inference attack
AUC: Area under curve
CPAR: Conditional probabilistic auto-regressive network
DTW: Dynamic time warping
EHR: Electronic health record
GAN: Generative adversarial network
GoF: Goodness-of-Fit
GRU: Gated recurrent unit
ICD-9: International classification of diseases-9
KS: Kolmogorov-Smirnov
LSTM: Long short-term memory
MAE: Mean absolute error
MIMIC-IV: Medical information mart for intensive Care-IV
RNN: Recurrent neural network
tSNE: t-distributed stochastic neighbour embedding
TSTR: Train synthetic test real
UMAP: Uniform manifold approximation and projection
VAE: Variational auto-encoder

## Footnotes

1 https://sdv.dev/

2 https://gretel.ai/synthetics

3 https://github.com/JimAchterbergLUMC/SynLongEHR

4 ICD-9 chapters can be found on the CDC website https://www.cdc.gov/nchs/icd/icd9cm.htm

5 Implementation available at https://synthetics.docs.gretel.ai/en/stable/models/timeseries_dgan.html

6 Implementation available at https://docs.sdv.dev/sdv/sequential-data/modeling/parsynthesizer
